# Prevalence of Gun Carrying and Gun Violence Victimization and Perpetration Among a Nationally Representative Sample of U.S. Youth and Young Adults

**DOI:** 10.1016/j.focus.2024.100294

**Published:** 2024-11-12

**Authors:** Bruce G. Taylor, Kimberly J. Mitchell, Heather A. Turner, Jackie Sheridan-Johnson, Elizabeth A. Mumford

**Affiliations:** 1 NORC at the University of Chicago, Bethesda, Maryland; 2 Crimes against Children Research Center (CCRC), University of New Hampshire, Durham, New Hampshire

**Keywords:** Firearms, guns, gun carrying, gun violence, victimization, perpetration, survey

## Abstract

**Introduction:**

Guns were one of the leading causes of death in children and youth aged 0–24 years in the U.S. over the last decade, with large variations by sex, race, region, and income. This paper examines the rates of gun involvement for a group of youth and young adults in the U.S. from ages 10 years to 34 years across major demographic and background factors.

**Methods:**

This is a cross-sectional nationally representative survey of participants recruited through an ongoing national research panel. Completed surveys were collected from 5,311 participants from September 2023 to January 2024.

**Results:**

Lifetime rates for carrying guns were 26.7%, gun violence victimization rates were 9.5%, and gun violence perpetration rates were 3.3%. High levels of past-year involvement with guns and gun violence were observed, with 13.9% carrying guns, gun violence victimization of 2.5%, and gun violence perpetration of 0.9%. Even more concerning, data indicate that 7.6% of the participants reported carrying a gun before age 18 years, 5.2% experienced gun violence victimization, and 1.8% perpetrated gun violence. Gun carrying and violence were higher by demographics, with a statistically significant difference for all demographic subgroups on at least 1 gun outcome.

**Conclusions:**

High levels of exposure to guns and gun violence are experienced among a nationally representative population of young Americans, with rates varying considerably by demographic factors. On the basis of these demographic disparities in gun involvement, more work is needed to address community and other risk and protective factors across different levels of the social ecology, such as structural inequities associated with gun violence.

## HIGHLIGHTS

• High levels of exposure to gun carrying and gun violence were found among young Americans.
• Gun carrying and gun violence rates vary considerably by demographic factors.
• This is most current nationally representative survey on young persons’ involvement with gun violence.

## INTRODUCTION

With 20,948 gun homicide deaths in 2021^1^ and an annual cost of $493.2 billion to the American economy,^2^ gun violence is a significant public health problem. The homicide rate in the U.S. is 7.5 times higher than the homicide rate in 28 other high-income countries combined, largely attributable to a 25 times higher gun homicide rate.^3^ In addition, from 2018 through 2021, an estimated 100,000 people experienced fatal or nonfatal gun injuries each year.^4^

Guns were the leading cause of death in children and youth aged 0–24 years in the U.S. in 2020,^5^ with important variations by region (e.g., homicide rates tend to be highest in the Southeast)^6^ and counties with lower income and higher poverty concentration.^7^^,^^8^ Gun homicides are concentrated in youth and young adults, men, and racial minority groups.^6^^,^^9^ There are significant disparities for gun involvement on the basis of race or ethniciity.^10^, ^11^, ^12^ Indeed, the largest increases in gun homicide rates between 2019 and 2020 were among non-Hispanic African American men aged 10–44 years and non-Hispanic American Indian or Alaska Native men aged 25–44 years.^9^ Men are more likely to own, use, kill with, and die by guns than women.^13^, ^14^, ^15^, ^16^ Adolescent males are 5 times more likely to carry a gun than women.^17^, ^18^, ^19^, ^20^ In addition, gun carrying increases with age during adolescence,^20^, ^21^, ^22^ and living in a rural area is associated with higher gun ownership.^14^ However, higher rates of youth gun homicides are found in urban areas.^23^

Nationally representative survey research is critical to an understanding of U.S. gun violence to gain a more nuanced understanding of the different forms of gun violence victimization and perpetration beyond the rates available through the National Vital Statistics System.^24^ However, such data are in very limited supply. This dearth of data stands in contrast to the collection of accurate annual health estimates for over 60 diseases.^25^^,^^26^ Although the National Crime Victimization Survey covers victimization, it does not cover gun violence perpetration or gun carrying.^27^ Other data sources for guns are found in a variety of national surveys (the General Social Survey, the Behavioral Risk Factor Surveillance System, and the National Longitudinal Surveys) but are brief measures used for monitoring health.^28^ Although these are all rigorous, they only have a few items on gun carrying and gun violence and little on perpetration. In addition, there are over 20 youth firearm studies with smaller nonrepresentative samples,^29^ but these are unlikely to provide generalizable findings nor cover the full range of gun-involved experiences.

This study aimed to provide prevalence estimates for different forms of gun carrying and gun violence among a U.S. nationally representative sample (aged 10–34 years). This paper examined the rates of gun carrying and gun violence victimization and perpetration and examined these rates across major demographic factors (sex, age, race/ethnicity, sexual identity, urbanicity, income, and region). The hypothesis of this study was that it would find significant demographic variations in gun involvement but recognizing that these measures were proxies for social and environmental contexts that increase risk and are not the risk factors themselves (to be investigated in later research). However, these variations were identified as a necessary first step in targeting intervention efforts.

## METHODS

### Study Sample

This study, Growing Up With Guns, analyzed a cross-sectional sample of 5,311 participants drawn from the AmeriSpeak panel (September 2023–January 2024) of over 60,000 U.S. residents. AmeriSpeak selects a stratified random sample of U.S. households using area probability and address-based sampling from the NORC National Sample Frame. These sampled households are then contacted by U.S. mail, telephone, and field interviewers (face to face) to capture harder-to-reach respondents. The AmeriSpeak household recruitment rate is 37%, one of the highest for similar national panels.^30^ AmeriSpeak covers about 97% of the U.S. household population. All analyses used data weighted to U.S. Census benchmarks, taking into account selection probabilities (balanced by sex, age, education, race or ethnicity, and region) and nonresponse (using a response propensity approach).^31^

Randomly selected AmeriSpeak panelists were sent an email invitation to complete this survey in English or Spanish. Participants not responding to the first invitation were contacted multiple times by email and phone. Participants who provided consent and completed the survey received an incentive worth $20. This study was approved by the research team's IRB and met the standards for protection of human subjects concerning their safety and privacy. Informed consent was obtained from all participants aged ≥18 years. For those aged <18 years, parental consent and child assent were obtained.

Completed surveys were received from 5,311 participants. Study eligibility included households with someone aged 10–34 years, living in the U.S., and being proficient in English or Spanish. The survey completion rate among those sampled was 62.5% for those aged 10–17 years and 29.1% for those aged 18–34 years. The participants were diverse with respect to a full set of demographic and background factors (Table 1).

**Table 1:** Weighted Sample Description (N=5,311)

Characteristic	*n* (%)
Birth sex	
Female	3,282 (49.6)
Male	1,908 (47.9)
Intersex	17 (0.3)
Missing	104 (2.3)
Sex identity	
Women	3,174 (47.4)
Men	1,870 (47.1)
Sex minority	166 (3.6)
Missing	101 (1.9)
Age, years	
10–17	1,189 (30.2)
18–25	853 (28.2)
26–34	3,269 (41.6)
Race/ethnicity	
White	2,803 (62.9)
Black or African American	946 (14.2)
Asian	535 (7.1)
American Indian or Alaska Native	63 (0.8)
Native Hawaiian	20 (0.4)
Other race	281 (4.1)
Two or more races	523 (7.5)
Missing	140 (3.0)
Hispanic ethnicity	1,104 (22.4)
Urbanicity	
Urban	2,260 (36.9)
Suburban	2,311 (48.8)
Rural	740 (14.3)
Sexual minority identity	1,047 (18.3)
Household Income	
<$30,000	1,273 (22.8)
$30,000 to <$60,000	1,373 (24.2)
$60,000 to <$100,000	1,260 (24.2)
≥$100,000	1,365 (28.0)
Missing	40 (0.9)
Region	
Northeast	640 (16.6)
Midwest	1,367 (20.3)
South	1,901 (38.6)
West	1,403 (24.6)

*Note:* Weighted percentages and unweighted *n* are presented.

### Measures

This study included measures of participants’ sex, age, race or ethnicity, sexual minority identity, urbanicity, household income, and study region (Table 1). Drawing from previous work,^32^^,^^33^ the study assessed gun carrying, gun violence victimization, and gun violence perpetration. The study asked about carrying a gun (possessing gun in hand, pocket, or in a bag), excluding gun carrying for hunting, target shooting, or a gun safety class. The study further asked about lifetime and past-year victimization (has been threatened with a gun, had a gun used to take something that you were carrying or wearing or something from your home while you were home, or was shot at by someone with a gun that had real bullets). The study had similar lifetime and past-year questions on perpetration (has threatened to hurt someone, take something away, or shot at someone with a gun that had real bullets). Furthermore, the study measured victimization and perpetration as minors by asking the same questions for all youth respondents aged <18 years (lifetime) and with reference to gun involvement before the age of 18 years for adult respondents (aged ≥18 years).

### Statistical Analysis

As seen in Table 2, Table 3, Table 4, weighted descriptive analyses and 3-weighted logistic regressions, calculated using Stata 18 software, were used to describe incidents of gun carrying, gun violence victimization, and gun violence perpetration. Within each of these 3 measures, the study has grouped the results into categories reflecting lifetime, past-year, and before-age-18-years (to distinguish childhood experiences in particular) experiences (recognizing that this is a conceptually different timeframe for adults to reflect on life before they were aged 18 years rather than the simpler ever or past-year measures). Subgroup differences were explored by demographics (as suggested by past research) to reduce Type I errors for each gun model. The study also limited Type I errors by observing that most of the significant results held up even with a lower critical value for each gun model.

**Table 2:** Frequencies and Adjusted Odds of Gun Carrying by Respondent Characteristics

		Prevalence			Adjusted odds		
		Lifetime	Past year	Before age 18 years	Lifetime	Past year	Before age 18 years
Overall	Group *n*	*n* (%)	*n* (%)	*n* (%)	OR (95% CI)	OR (95% CI)	OR (95% CI)
All participants	5,311	1,402 (25.7)	767 (13.9)	379 (7.6)			
Sex identity							
Women	3,174	756 (20.5)	395 (10.3)	175 (5.5)	1.0 (ref)	1.0 (ref)	1.0 (ref)
Men	1,870	583 (31.0)	339 (17.6)	177 (9.1)	1.9 (1.6, 2.3)***	2.0 (1.6, 2.5)***	1.6 (1.2, 2.2)***
Sex minority	166	33 (21.4)	16 (10.3)	15 (11.9)	1.2 (0.7, 2.1)	1.2 (0.6, 2.4)	2.5 (1.0, 6.2)*
Age, years							
10–17	1,189	98 (7.5)	35 (2.5)	—	1.0 (ref)	1.0 (ref)	1.0 (ref)
18–25	853	236 (29.4)	132 (15.6)	73 (8.3)	6.8 (4.8, 9.7)***	9.2 (5.5, 15.4)***	1.4 (0.9, 2.0)
26–34	3,269	1,068 (36.5)	600 (21.1)	208 (7.2)	9.1 (6.7, 12.4)***	13.3 (8.3, 21.3)***	1.1 (0.8, 1.5)
Race/Ethnicity							
White	2,803	722 (25.2)	374 (13.7)	181 (6.5)	1.0 (ref)	1.0 (ref)	1.0 (ref)
Black/African-American	946	334 (34.7)	212 (20.0)	86 (11.9)	1.7 (1.4, 2.3)***	1.8 (1.3, 2.4)***	2.4 (1.6, 3.7)***
Asian	535	80 (16.3)	29 (6.2)	21 (5.0)	0.5 (0.3, 0.7)***	0.4 (0.2, 0.7)***	0.8 (0.4, 1.6)
American Indian/ Alaska Native	63	25 (45.1)	17 (25.4)	11 (22.7)	2.2 (0.8, 6.0)	1.9 (0.8, 4.1)	3.2 (1.1, 9.1)*
National Hawaiian	20	6 (31.6)	4 (25.2)	2 (9.6)	1.3 (0.3, 4.6)	2.2 (0.5, 10.7)	1.2 (0.3, 5.1)
≥2 races	523	133 (24.3)	77 (11.9)	45 (8.7)	0.9 (0.6, 1.3)	0.8 (0.6, 1.2)	1.2 (0.8, 2.0)
Hispanic ethnicity							
No	4,149	1,094 (26.1)	600 (14.0)	266 (6.9)	1.0 (ref)	1.0 (ref)	1.0 (ref)
Yes	1,104	299 (25.5)	163 (14.1)	111 (10.1)	1.1 (0.9, 1.5)	1.2 (0.8, 1.6)	1.6 (1.1, 2.4)**
Urbanicity							
Urban	2,260	551 (23.9)	288 (12.1)	160 (8.1)	1.0 (ref)	1.0 (ref)	1.0 (ref)
Suburban	2,311	602 (24.4)	330 (13.2)	145 (5.9)	1.1 (0.9, 1.4)	1.1 (0.9, 1.5)	0.8 (0.6, 1.2)
Rural	740	249 (35.1)	149 (21.2)	74 (11.7)	2.2 (0.6, 1.1)	2.3 (1.7, 3.3)***	2.1 (1.3, 3.3)**
Sexual identity							
Heterosexual	4,264	1,139 (25.9)	641 (14.3)	298 (7.5)	1.0 (ref)	1.0 (ref)	1.0 (ref)
LGBQA+	1,047	263 (25.0)	126 (12.5)	81 (7.9)	0.9 (0.7, 1.1)	0.8 (0.6, 1.0)	1.0 (0.6, 1.6)
Household income							
<$30,000	1,273	428 (30.9)	248 (17.3)	128 (9.2)	1.0 (ref)	1.0 (ref)	1.0 (ref)
$30,000 to <$60,000	1,373	354 (24.1)	188 (12.7)	89 (6.5)	0.8 (0.6, 1.0)*	0.7 (0.5, 1.0)*	0.8 (0.6, 1.2)
$60,000 to <$100,000	1,260	365 (30.8)	202 (16.5)	93 (8.7)	1.3 (1.0, 1.7)*	1.2 (0.9, 1.7)	1.2 (0.8, 1.7)
≥$100,000	1,365	251 (19.1)	129 (10.5)	65 (6.1)	0.8 (0.6, 1.1)	0.9 (0.6, 1.2)	0.9 (0.6, 1.4)
Region							
Northeast	640	130 (20.9)	60 (10.2)	29 (5.1)	1.0 (ref)	1.0 (ref)	1.0 (ref)
Midwest	1,367	347 (25.8)	192 (13.9)	110 (7.8)	1.3 (0.9, 1.8)	1.4 (0.9, 2.1)	1.3 (0.7, 2.4)
South	1,901	627 (30.5)	379 (18.1)	139 (7.9)	1.7 (1.2, 2.3)***	1.9 (1.3, 2.8)**	1.4 (0.8, 2.5)
West	1,403	298 (21.6)	136 (9.9)	101 (8.5)	1.3 (0.9, 1.8)	1.1 (0.7, 1.7)	1.9 (1.0, 3.5)*
McFadden's pseudo R squared test					0.13	0.14	0.10

*Note*: *p<0.05, **p<0.01, and ***p<0.001.

LGBQA, lesbian, gay, bisexual, questioning, or asexual.

**Table 3:** Frequencies and Adjusted Odds of Gun Victimization by Respondent Characteristics

		Prevalence			Adjusted odds		
		Lifetime	Past year	Before age 18 years	Lifetime	Past year	Before age 18 years
Overall	Group *n*	*n* (%)	*n* (%)	*n* (%)	OR (95% CI)	OR (95% CI)	OR (95% CI)
All participants	5,311	595 (9.5)	149 (2.5)	301 (5.2)	—	—	—
Sex identity							
Women	3,174	351 (8.5)	75 (1.9)	174 (4.8)	1.0 (ref)	1.0 (ref)	1.0 (ref)
Men	1,870	204 (9.8)	62 (2.7)	106 (4.9)	1.3 (1.0, 1.6)*	1.5 (0.9, 2.2)	1.0 (0.7, 1.5)
Sex minority	166	28 (17.1)	6 (4.7)	16 (11.5)	2.0 (1.0, 3.8)*	2.1 (0.6, 8.1)	2.1 (0.9, 4.8)
Age, years							
10–17	1,189	39 (2.5)	21 (1.2)	—	1.0 (ref)	1.0 (ref)	1.0 (ref)
18–25	853	103 (10.1)	35 (3.5)	63 (6.7)	3.5 (2.1, 5.9)***	2.1 (1.1, 4.3)*	2.2 (1.3, 3.7)**
26–34	3,269	453 (14.0)	93 (2.7)	199 (6.0)	5.5 (3.5, 8.8)***	2.0 (1.1, 3.6)*	2.1 (1.3, 3.4)**
Race/ethnicity							
White	2,803	254 (8.2)	57 (1.7)	124 (4.2)	1.0 (ref)	1.0 (ref)	1.0 (ref)
Black/African American	946	154 (14.6)	42 (4.4)	72 (7.9)	1.7 (1.2, 2.4)**	2.0 (1.1, 3.5)**	1.7 (1.1, 2.7)*
Asian	535	30 (4.7)	6 (1.5)	16 (2.6)	0.5 (0.3, 0.9)*	0.9 (0.2, 3.7)	0.6 (0.2, 1.5)
American Indian/Alaska Native	63	14 (14.2)	4 (4.8)	9 (9.8)	1.3 (0.6, 2.7)	2.5 (0.6, 9.9)	1.9 (0.8, 4.7)
National Hawaiian	20	5 (28.8)	2 (20.0)	1 (4.9)	3.1 (0.8, 11.8)	13.1 (2.8, 60.9)***	0.9 (0.1, 7.4)
≥2 races	523	93 (13.2)	26 (5.1)	52 (8.5)	1.6 (1.1, 2.3)*	3.1 (1.6, 5.9)***	1.9 (1.1, 3.1)**
Hispanic ethnicity							
No	4,149	461 (9.3)	116 (2.5)	235 (5.1)	1.0 (ref)	1.0 (ref)	1.0 (ref)
Yes	1,104	130 (10.3)	32 (2.5)	66 (5.7)	1.1 (0.7, 1.5)	0.9 (0.5, 1.7)	0.9 (0.6, 1.5)
Urbanicity							
Urban	2,260	266 (10.4)	67 (2.8)	132 (5.6)	1.0 (ref)	1.0 (ref)	1.0 (ref)
Suburban	2,311	231 (8.3)	58 (2.2)	122 (4.7)	0.9 (0.7, 1.3)	1.1 (0.7, 1.7)	1.0 (0.7, 1.4)
Rural	740	98 (11.0)	24 (2.6)	47 (5.7)	1.3 (0.9, 1.8)	1.1 (0.6, 2.1)	1.2 (0.8, 2.0)
Sexual identity							
Heterosexual	4,264	439 (8.5)	116 (2.3)	223 (4.6)	1.0 (ref)	1.0 (ref)	1.0 (ref)
LGBQA+	1,047	156 (13.7)	33 (3.5)	78 (7.6)	1.4 (1.0, 1.9)*	1.2 (0.7, 2.1)	1.3 (0.9, 1.9)
Household Income							
<$30,000	1,273	217 (13.3)	65 (4.0)	104 (7.0)	1.0 (ref)	1.0 (ref)	1.0 (ref)
$30,000 to <$60,000	1,373	179 (12.3)	41 (2.9)	94 (6.9)	1.0 (0.7, 1.4)	0.8 (0.5, 1.3)	1.2 (0.8, 1.8)
$60,000 to <$100,000	1,260	113 (7.7)	23 (1.5)	56 (3.7)	0.7 (0.5, 0.9)**	0.5 (0.2, 0.9)**	0.6 (0.4, 1.0)*
≥$100,000	1,365	86 (5.7)	20 (1.9)	47 (3.5)	0.6 (0.4, 0.8)**	0.6 (0.3, 1.3)	0.7 (0.4, 1.2)
Region							
Northeast	640	60 (6.5)	10 (1.3)	25 (2.8)	1.0 (ref)	1.0 (ref)	1.0 (ref)
Midwest	1,367	149 (8.8)	38 (2.5)	78 (4.7)	1.3 (0.9, 2.0)	1.6 (0.7, 4.1)	1.6 (0.8, 3.0)
South	1,901	229 (10.8)	62 (3.1)	111 (5.8)	1.5 (1.0, 2.3)*	1.9 (0.7, 4.8)	2.0 (1.1, 3.8)*
West	1,403	157 (9.9)	39 (2.4)	87 (6.1)	1.6 (1.1, 2.5)*	1.8 (0.7, 4.7)	2.3 (1.2, 4.6)**
McFadden's pseudo R squared test					0.10	0.10	0.10

*Note*: *p<0.05, **p<0.01, and ***p<0.001.

LGBQA, lesbian, gay, bisexual, questioning, or asexual.

**Table 4:** Frequencies and Adjusted Odds of Gun Perpetration by Respondent Characteristics

		Prevalence			Adjusted odds		
		Lifetime	Past year	Before age 18 years	Lifetime	Past year	Before age 18 years
Overall	Group *n*	*n* (%)	*n* (%)	*n* (%)	OR (95% CI)	OR (95% CI)	OR (95% CI)
All participants	5,311	197 (3.3)	50 (0.9)	84 (1.8)	—	—	—
Sex identity							
Women	3,174	99 (2.3)	25 (0.6)	42 (1.2)	1.0 (ref)	1.0 (ref)	1.0 (ref)
Men	1,870	77 (4.0)	17 (0.8)	30 (2.0)	1.9 (1.3, 2.9)***	1.3 (0.6, 2.9)	1.9 (1.0, 3.4)*
Sex minority	166	9 (3.8)	1 (0.6)	5 (1.6)	1.6 (0.7, 3.9)	0.9 (0.1, 7.3)	1.0 (0.3, 3.3)
Age, years							
10–17	1,189	26 (1.8)	6 (0.5)	—	1.0 (ref)	1.0 (ref)	1.0 (ref)
18–25	853	37 (3.7)	13 (1.4)	21 (2.5)	1.8 (0.9, 3.9)	1.6 (0.4, 6.4)	1.0 (0.4, 2.6)
26–34	3,269	134 (4.2)	31 (0.8)	37 (1.2)	2.3 (1.2, 4.5)**	1.3 (0.4, 4.4)	0.6 (0.2, 1.3)
Race/ethnicity							
White	2,803	69 (2.3)	14 (0.5)	26 (0.9)946	1.0 (ref)	1.0 (ref)	1.0 (ref)
Black/African American	946	62 (6.1)	14 (1.8)	30 (3.7)	2.5 (1.6, 3.9)***	3.0 (1.1, 8.0)*	3.5 (1.8, 7.1)***
Asian	535	11 (2.9)	5 (0.8)	6 (2.0)	1.3 (0.5, 3.5)	3.3 (0.7, 15.0)	2.9 (0.7, 11.9)
Amer Indian/Alaska Native	63	4 (15.2)	1 (1.9)	2 (10.0)	6.5 (1.5, 27.9)**	3.1 (0.4, 22.9)	12.7 (1.9, 84.2)**
National Hawaiian	20	2 (5.0)	1 (0.6)	0	1.9 (0.2, 15.3)	0.7 (0.1, 8.7)	-
≥2 races	523	35 (7.7)	9 (2.4)	15 (5.0)	3.3 (1.6, 6.9)***	3.7 (1.3, 10.7)**	5.0 (1.7, 14.7)**
Hispanic ethnicity							
No	4,149	142 (3.1)	30 (0.7)	58 (1.5)	1.0 (ref)	1.0 (ref)	1.0 (ref)
Yes	1,104	52 (4.2)	20 (1.7)	24 (2.5)	1.5 (0.9, 2.5)	4.2 (1.7, 10.3)**	1.9 (0.8, 4.3)
Urbanicity							
Urban	2,260	91 (3.3)	24 (0.7)	37 (1.4)	1.0 (ref)	1.0 (ref)	1.0 (ref)
Suburban	2,311	79 (3.5)	22 (1.1)	37 (2.2)	1.3 (0.9, 2.0)	2.3 (.1, 5.0)*	2.2 (1.2, 4.0)**
Rural	740	27 (3.2)	4 (0.6)	10 (1.3)	1.3 (0.7, 2.3)	1.2 (0.3, 4.7)	1.2 (0.4, 3.4)
Sexual identity							
Heterosexual	4,264	146 (3.2)	42 (0.9)	59 (1.7)	1.0 (ref)	1.0 (ref)	1.0 (ref)
LGBQA+	1,047	51 (3.9)	8 (0.9)	25 (2.2)	1.2 (0.7, 2.1)	1.2 (0.4, 3.5)	1.6 (0.7, 3.9)
Household income							
<$30,000	1,273	71 (4.5)	23 (1.4)	32 (2.3)	1.0 (ref)	1.0 (ref)	1.0 (ref)
$30,000 to <$60,000	1,373	55 (3.1)	10 (0.7)	24 (1.7)	0.7 (0.5, 1.2)	0.6 (0.2, 2.0)	0.8 (0.4, 1.7)
$60,000 to <$100,000	1,260	43 (3.7)	9 (0.9)	16 (2.0)	1.0 (0.6, 1.8)	1.0 (04, 2.5)	1.0 (0.4, 2.2)
≥$100,000	1,365	27 (2.3)	8 (0.6)	11 (1.1)	0.7 (0.4, 1.3)	0.6 (0.2, 1.9)	0.5 (0.2, 1.1)
Region							
Northeast	640	17 (2.3)	4 (0.6)	9 (1.3)	1.0 (ref)	1.0 (ref)	1.0 (ref)
Midwest	1,367	50 (3.1)	17 (1.3)	27 (1.9)	1.7 (0.8, 3.6)	2.5 (0.6, 10.8)	1.6 (0.6, 4.3)
South	1,901	89 (4.4)	22 (1.1)	32 (2.1)	2.1 (1.0, 4.3)*	2.0 (0.5, 8.3)	1.7 (0.6, 4.4)
West	1,403	41 (2.7)	7 (0.3)	16 (1.4)	1.3 (0.5, 3.1)	0.4 (0.1, 2.2)	0.9 (0.2, 3.5)
McFadden's pseudo R squared test					0.07	0.10	0.10

*Note*: *p<0.05, **p<0.01, and ***p<0.001.LGBQA, lesbian, gay, bisexual, questioning, or asexual.

Only the significant results are presented in the next section in the order of presentation in Table 2, Table 3, Table 4. Model fit was acceptable in all the models on the basis of McFadden's pseudo-R squared test (results presented in the last row of each logistic regression table).^34^

## RESULTS

**Figure 1:**
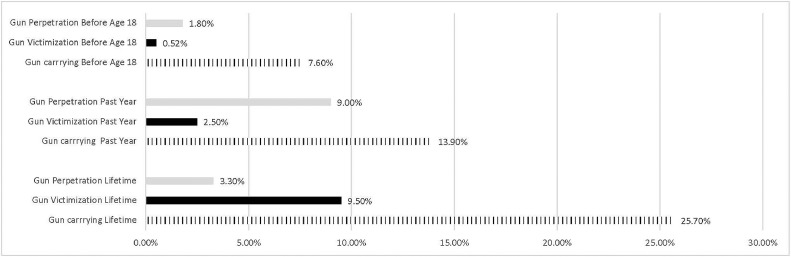
Weighted prevalence for gun carrying, gun victimization, and gun perpetration by experience before age 18 years, past-year experience, and lifetime experience.

As seen in Figure 1 and Table 2, 25.7% of all participants reported having carried a gun in their lifetime, 13.9% reported having carried a gun in the past year, and 7.6% reported having carried a gun before the age of 18 years. On the basis of the logistic regression model in Table 2, men had higher rates of gun carrying than women (lifetime OR=1.9, *p*<0.001; past-year OR=2.0, *p*<0.001; before age-18-years OR=1.6, *p*<0.001). Participants aged 18–25 years (lifetime OR=6.8, *p*<0.001; past-year OR=9.2, *p*<0.001) and participants aged 26–34 years (lifetime OR=9.1, *p*<0.001; past-year OR=13.3, *p*<0.001) reported higher gun carrying rates than participants aged 10–17 years. African-Americans (lifetime OR=1.7, *p*<0.001; past-year OR=1.8, *p*<0.001; before-age-18-years OR=2.4, *p*<0.001) and American Indian or Alaska Natives (before-age-18 years; OR=3.2, *p*<0.05) had higher rates of gun carrying than those who identified as White, but Asian participants had lower rates (lifetime OR=0.5, *p*<0.001 and past- year OR=0.4, *p*<0.001). Hispanic participants had higher rates of gun carrying before age 18 years (OR=1.6, *p*<0.01). Rural participants had higher rates of gun carrying (past-year OR=2.3, *p*<0.001; before-age-18 years OR=2.1, *p*<0.01). Those participants reporting household income of $60,000 up to $100,000 had higher rates of gun carrying than participants reporting household income under $30,000 (lifetime measure OR=1.3, *p*<0.05). Participants from the South (lifetime OR=01.7, *p*<0.001; past-year OR=1.9, *p*<0.01) and the West (before-age-18-years OR=1.9, *p*<0.05) had higher rates of gun carrying than participants from the Northeast.

As seen in Figure 1 and Table 3, 9.5% of all participants reported having experienced gun violence victimization in their lifetime, 2.5% reported having experienced gun violence victimization in the past year, and 5.2% reported having experienced gun violence victimization before the age of 18 years. On the basis of the logistic regression model in Table 3, men (lifetime OR=1.3, *p*<0.05) and those who do not identified as a man nor a woman (sex minority group) (lifetime OR=2.0, *p*<0.05) experienced higher rates of gun violence victimization than women. Participants aged 18–25 years (lifetime OR=3.5, *p*<0.001; past-year OR=2.1, *p*<0.05; before-age-18-years OR=2.2, *p*<0.01) and those aged 26–34 years (lifetime OR=5.5, *p*<0.001, past-year OR=2.0, *p*<0.05; before-age-18-years OR=2.1, *p*<0.01) reported higher gun violence victimization rates than participants aged 10–17 years. African-Americans (lifetime OR=1.7, *p*<0.01; past-year OR=2.0, *p*<0.01; before-age-18-years OR=1.7, *p*<0.05), Native Hawaiians (past-year OR=3.1, *p*<0.001), and multiracial participants (lifetime OR=1.6, *p*<0.05; past-year OR=3.1, *p*<0.001; before-age-18-years OR=1.9, *p*<0.01) had higher rates of gun violence victimization than those who identified as White, but Asians reported lower rates (lifetime OR=0.5, *p*<0.05) than Whites. Participants who identified as lesbian, gay, bisexual, questioning, or asexual (LGBQA+) reported higher rates of gun violence victimization rates than heterosexuals (lifetime OR=1.4, *p*<0.05). Participants reporting household income of $60,000 up to $100,000 (lifetime OR=0.7; *p*<0.01; past-year OR=0.5, *p*<0.01; before-age-18-years OR=0.6, *p*<0.05) and $100,000 or more had lower rates of gun violence victimization rates (lifetime OR=0.6, *p*<0.01) than those under $30,000. Participants from the South (lifetime OR=1.5, *p*<0.05; before-age-18-years OR=2.0, *p*<0.05) and West (lifetime OR=1.6, *p*<0.05; before-age-18-years OR=2.3, *p*<0.01) had higher rates of gun violence victimization than those from the Northeast.

As seen in Figure 1 and Table 4, 3.3% of all participants self-reported perpetrating gun violence in their lifetime, 0.9% self-reported perpetrating gun violence in the past year, and 1.8% self-reported perpetrating gun violence before the age of 18 years. On the basis of the logistic regression model in Table 4, men had higher rates of gun violence perpetration than women (lifetime OR=1.9, *p*<0.001 and before-age-18-years OR=1.9, *p*<0.05). Those aged 26–34 years had higher lifetime gun violence perpetration rates (OR=2.3, *p*<0.001) than those aged 10–17 years. African-Americans (lifetime OR=2.5, *p*<0.001; past-year OR=3.0, *p*<0.05; before-age-18-years OR=3.5, *p*<0.001), American Indian or Alaska Native (lifetime OR=6.5, *p*<0.001; before-age-18-years OR=12.7, *p*<0.01), and multiracial participants (lifetime OR=3.3, *p*<0.001; past-year OR=3.7, *p*<0.01; before-age-18-years OR=5.0, *p*<0.01) reported higher gun violence perpetration than participants who identified as White. Those who identified as Hispanic reported higher rates of past-year gun violence perpetration than non-Hispanics (OR=4.2, *p*<0.01). Suburban participants reported higher rates of gun violence perpetration than those in urban areas (past-year OR=2.3, *p*<0.05; before-age-18-years OR=2.2, *p*<0.01). Participants from the South reported higher rates of gun violence perpetration than participants from the Northeast (lifetime OR=2.1, *p*<0.05).

## DISCUSSION

The Growing Up With Guns study offers one of the first nationally representative surveys focused on gun violence among youth and young adults. In this sample, the study found high levels of gun carrying and gun violence. With 112,181,498 Americans aged 10–34 years as of 2023 U.S. Census data,^35^ this translates into nearly 29 million Americans aged 10–34 years carrying guns in their lifetime (on the basis of a 25.7% rate from the present study's findings), 10.66 million victims of gun violence (9.5%), and 3.7 million perpetrators of gun violence (3.3%). Even more concerning, this study's data indicate that millions of minors in America are involved with gun violence. With 34,437,013 Americans between the ages 10 years and 17 years,^35^ the study extrapolated that 2.6 million Americans are carrying a gun before the age of 18 years (on the basis of a 7.6% rate from this study's findings); 1.8 million Americans aged 10–17 years are victims of gun violence (5.2%); and 620,000 Americans aged 10–17 years have perpetrated gun violence (1.8%).

The study also uncovered that gun carrying and gun violence were higher for certain groups. The authors found statistically significant differences for all demographic subgroups on at least 1 of the study outcomes. However, it was found that there were fewer distinctions for gun violence perpetration by demographic factors than for gun carrying and victimization, perhaps because of potential under-reporting of perpetration at only 1.8% compared with 5.2% for victimization.^36^ However, the authors recognize that although smaller in number, a few perpetrators may account for a larger percentage of victimizations by engaging in gun violence perpetration multiple times. This is an area for further research.

First, men had significantly higher rates of gun carrying, gun victimization, and gun perpetration. This result is explainable because men^13^ are almost twice as likely to own guns as women.^14^ Those around guns are also more likely to be victims of and perpetrate gun violence.^37^^,^^38^ The results are consistent with research showing men to be more likely to be both the victims and perpetrators of gun violence,^15^^,^^16^ with the exception of intimate partner violence, through which women are more than twice as likely as men to be victims of gun violence.^39^ Understanding these gendered patterns is an important area for further research. In addition, it suggests the need for distinct culturally competent messaging for gun safety for men and women. Women who use guns, a rarer group, may have different concerns regarding self-protection outside the home, for example, than men. Firearms are the leading mechanism of suicide among U.S. women,^40^ and the unique needs of women should be addressed in suicide-prevention messaging. Current suicide prevention efforts generally focus on men, given the higher proportion of gun ownership among men, as well as suicide in military and veteran populations, which are also predominantly men.^40^

Overall, the youngest participants (aged <18 years) were the least likely to carry a gun and be a victim or perpetrator of gun violence. These findings are consistent with earlier research that shows that gun carrying increases during adolescence.^20^^,^^21^ Those aged 17–24 years are overrepresented in homicides involving guns,^12^^,^^41^ with the gun mortality rates of youths aged 15–24 years more than 10 times as high as those for children aged 10–14 years.^22^ A 2021 Centers for Disease Control and Prevention study found the highest gun homicide victimization rates among those ages 25–44 years.^42^ Lower involvement with guns among those aged <18 years highlights the opportunity to advance primary prevention with youth, because they are not already typically involved in gun violence. Interventions with youth aged <18 years, based on risk factors associated with gun violence seen among young adults, can help develop an understanding of the risks associated with guns and build skillsets to avoid gun violence in young adulthood.

The study found significant differences in gun carrying and gun violence involvement by race or ethnicity. The results of this study are largely consistent with the literature that shows significant disparities for gun involvement based on race. For example, gun homicide rates among Black children and adolescents are approximately 2–4 times higher than those among Latinx and indigenous peers and 10–14 times higher than those among White and Asian American peers.^12^ Gun homicide rates are also elevated for indigenous young men in comparison with those for White and Asian youth.^43^ To address the higher rates of gun carrying and gun violence victimization and perpetration among African-American or American Indian or Alaska Native youth and young adults, there is a need for better community-level interventions to address the environmental and social factors that serve as precursors to gun violence within these ethnic groups.^37^^,^^44^ These factors can shape the perception that gun possession and violence are the best survival tools available in certain communities.^37^ There is a need to address systemic inequalities, such as poverty and educational disparities, which contribute to the prevalence of gun violence.^45^ By implementing policies that address these root causes and by providing resources for community development that provide pathways out of violence for different ethnic groups, it can create more equitable and safer environments for everyone.^37^

Those who identify as LGBQA+ had higher rates of gun violence victimization rates than those who identify as heterosexual for the lifetime measure. The study did not find any literature that covered this issue. However, given the higher rates of violence more generally faced by youth who identify as LGBQA+, this finding is consistent with that literature.^46^, ^47^, ^48^ Victim service organizations should take notice of this finding and be prepared to offer culturally competent support services for LGBQA+ individuals and communities because they are overrepresented in the current estimate of gun violence victimization.

Consistent with the literature,^14^ the study found that rural participants reported gun carrying at higher rates than urban participants. However, the finding that suburban dwellers had higher rates of gun violence perpetration than urban dwellers were unexpected; several studies have reported higher rates of youth gun homicides and assaults in urban neighborhoods.^23^ The authors join the call for more research on gun violence in the suburbs and the possible need for more gun violence prevention efforts in the suburbs that are often lagging behind urban localities in this area.^49^

Consistent with the literature that youth and young adults living in counties with lower income and higher poverty concentration have higher rates of unintentional injury-related death,^7^^,^^8^ it was also found that those with higher household income had lower rates of gun violence victimization than those with lower income. This finding underscores the need for additional services and prevention programming in low-income communities but also the need for education around safe gun storage in higher-income communities because, in alignment with the literature,^50^ it was found that participants with higher household income had higher rates of gun carrying than those with lower income.

These findings also point to the need for expanded prevention efforts and education on safe gun storage and carrying practices in the Southern U.S. In the present study, youth and young adults from this region had higher rates of gun carrying, gun violence victimization, and perpetration than those from the Northeast. These results are generally consistent with the finding in the literature of higher rates of gun ownership and gun homicides in the South, with fewer gun homicides in the Northeast or New England and rural Northwest.^6^

The findings support the need for training for those who work with young people (e.g., counselors and teachers) on how to identify signs of the potential for gun violence. In addition, there is a need for prevention programs to specifically target young adults (not just adolescents) to help better inform them of their greater risk for gun violence involvement. With the youngest youth in this study the least likely to be involved in gun violence, this creates an opportunity for more primary prevention with younger youth. Preventionists should work with social media organizations regarding their role in youth gun violence.^51^ Communities should engage their healthcare providers in pediatric settings in educating families about gun safety and in preventing youth gun violence.^52^ On the basis of the disparities that were found across demographic factors, it suggests that more work is needed to address community^53^ and other factors across different levels of the social ecology, such as structural inequities,^54^ associated with gun violence.

### Limitations

Although confidential self-report surveys have become an accepted modality for collecting youth violence data,^55^ the data are self-reported and thus are susceptible to possible under-reporting and social desirability. In that way, the results might be considered conservative estimates, with the true estimates possibly higher for gun carrying and gun violence involvement. The study also achieved a modest response rate, especially for the grouped aged 18–34 years (29.1%). This means that nonresponse bias could be affecting the results, and the study might be under-representing the experiences of some young people (e.g., those more likely to engage in gun violence). However, the study addressed this concern through the use of nonresponse weights, on the basis of observed demographic factors. Furthermore, the data for this study are cross-sectional and do not support causal analyses of relationships.

## CONCLUSIONS

Despite these limitations, this study advances the field, providing nationally representative data documenting the high levels of exposure to guns and gun violence among young persons. Concerns about gun carrying and gun violence are even higher for certain demographic groups. In summary, more work is needed to better identify when, with whom, and how to intervene to reduce gun violence.
